# Lower-Sized Chitosan Nanocapsules for Transcutaneous Antigen Delivery

**DOI:** 10.3390/nano8090659

**Published:** 2018-08-26

**Authors:** Juan I. Bussio, Carla Molina-Perea, José Vicente González-Aramundiz

**Affiliations:** 1 Departamento de Farmacia, Facultad de Química, Pontificia Universidad Católica de Chile, Santiago 7820436, Chile; jibussio@uc.cl (J.I.B.); cfmolina1@uc.cl (C.M.-P.); 2 Centro de Investigación en Nanotecnología y Materiales Avanzados “CIEN-UC”, Pontificia Universidad Católica de Chile, Santiago 7820436, Chile

**Keywords:** nanocapsules, chitosan, antigen delivery, transcutaneous immunization, vaccine

## Abstract

Transcutaneous vaccination has several advantages including having a noninvasive route and needle-free administration; nonetheless developing an effective transdermal formulation has not been an easy task because skin physiology, particularly the stratum corneum, does not allow antigen penetration. Size is a crucial parameter for successful active molecule administration through the skin. Here we report a new core-shell structure rationally developed for transcutaneous antigen delivery. The resulting multifunctional carrier has an oily core with immune adjuvant properties and a polymeric corona made of chitosan. This system has a size of around 100 nm and a positive zeta potential. The new formulation is stable in storage and physiological conditions. Ovalbumin (OVA) was used as the antigen model and the developed nanocapsules show high association efficiency (75%). Chitosan nanocapsules have high interaction with the immune system which was demonstrated by complement activation and also did not affect cell viability in the macrophage cell line. Finally, ex vivo studies using a pig skin model show that OVA associated to the chitosan nanocapsules developed in this study penetrated and were retained better than OVA in solution. Thus, the physicochemical properties and their adequate characteristics make this carrier an excellent platform for transcutaneous antigen delivery.

## 1. Introduction

Classical administration of vaccines is still parenteral (either intradermal, subcutaneous, or intramuscular). These administration routes have important disadvantages such as reduced patient compliance, the requirement of trained personnel, and potential safety risks (e.g., needle stick accidents, needle reuse, and wound infection) [1,2]. In addition, WHO estimates that 4–6 billion injections are unsafe in four of six regions of the world due to needle reuse, use of unsterilized needles, dangerous infections, etc. [3]. Needle-free vaccine delivery is desirable for many reasons. In fact, most descriptions of an ideal vaccine include a needle-free administration. This type of administration has the potential to lead to the following significant advances in immunization delivery: improved safety for the handler of the vaccine; the vaccine itself; and the community: better compliance with immunization schedules; decreased or eliminated injection site pain; easier and faster vaccine delivery; and reduced cost [4,5]. In this challenge of finding new administration routes, especially in vaccine delivery, skin seems to be a valid and interesting approach.

The skin is the most extensive organ in the human body and has a large number of immunocompetent and antigen presenting cells such as Langerhans cells, keratinocytes, dermal dendritic cells, macrophages, mast cells, and T cells in its composition [6]. This high density of immune cells suggests that transcutaneous vaccination promotes the induction of humoral and cellular immunity. Moreover, vaccine delivery through the skin might elicit mucosal immune responses at the site of virus entry and improve better cellular immunity, thus improving vaccine effectiveness [7,8]. However, the administration of vaccines through the skin is not an easy task; especially because the skin is an excellent barrier against outside agents. Structurally the skin has three layers (epidermis, dermis, and hypodermis), and within the epidermis, there are different layers, the first of which and the most important in the context of transdermal administration being the stratum corneum (SC). This stratum is a real barricade both literally and figuratively. On one hand it protects us from ultraviolet light, pathogenic microorganisms, chemical compounds, and others [9], but on the other hand, the SC is the principal challenge that needs to be overcome for successful transcutaneous vaccination [10]. 

Several approaches for overcoming or circumventing the SC have been studied among which devices such as microneedles, jet injectors, and electroporation stand out. It should be noted that these methods still damage the skin [11]. Another proposal for transcutaneous vaccination is the use of nanoparticles. The small size and their possibility to act as an adjuvant make nanoparticles an interesting approach regarding transdermal antigen delivery [12,13]. Nanoparticles are solid colloidal particles of a size that ranges from 1–1000 nm in diameter. Polymeric nanoparticles have applications in diagnostics and drug delivery because they can control the release of the molecule that they carry, they can combine therapy and imaging (theranostics); protection of drug molecules and their specific targeting areas; facilitate improvements in the therapeutic index [14]. Among polymeric nanoparticles, we can find emulsion droplets, nanosphere, nanocapsules, etc.

Polymeric nanocapsules are composed of an oily core which enables the encapsulation of hydrophobic active molecules and a polymer corona. This shell can increase their colloidal stability, control the release of the encapsulated drugs, and be used as targeting and transport hydrophilic molecules [15]. Materials such as polymers, polypeptides, polyaminoacids, and others have been studied as a nanocapsules shell [16,17]. Chitosan nanocapsules have been studied for cancer, infectious diseases, and vaccine development [18,19,20], in particular, chitosan opens tight junctions of transient epithelial cells and improves the transdermal permeation of different anti-inflammatory medications, hormones, and other delicate molecules [21,22,23].

Our main goal was the development of chitosan nanocapsules for transcutaneous vaccination. As size is a critical parameter for SC penetration, our experimental design allows nanocarriers with an oil-core structure surrounded by a chitosan shell of a lower size. We investigated the ovalbumin association as a model antigen and its immune system interaction (with cytotoxicity and complement activations studies). Finally, ex vivo studies show the ability of the new nanocapsules to penetrate and retained in the skin when compared to the model antigen in solution.

## 2. Materials and Methods 

### 2.1. Chemicals

Lipocol HCO-40 (LP-HCO) and Vitamin E or α tocopherol (TCPH) were kindly donated by Vantage (Chicago, IL, USA), Emulphor FAS-30 (EmFas-30) was purchased from BASF (Ludwigshafen, Germany). Ultrapure Chitosan hydrochloride salt (CS: Protasan UP CL 113, Mw: 125 kDa, deacetylation degree 86%) was obtained from Novamatrix (Sandika, Norway). Ovalbumin (OVA) was purchased from Invivogen (San Diego, CA, USA). The organic solvents HPLC grade (ethanol, methanol, acetonitrile, and acetone), Tris(hydroxymethyl)aminomethane (Tris), glycine, tween 20 and glycerol were purchased from Merck (Darmstadt, Germany). Tetramethylethylenediamine (TEMED), ammonium persulfate (APS) and Bromophenol blue were obtained from Amresco (Solon, OH, USA). 30% Acrylamide/Bis solution, Clarity™ Western ECL Substrate, Precision Plus Protein™ All Blue, Goat Anti-Rabbit IgG horseradish peroxidase conjugate and Goat Anti-mouse IgG horseradish peroxidase conjugate were supplied by Bio-rad (Hercules, CA, USA). RPMI, Penicillin-streptomycin solution, MEM eagle solution, DPBS, and fetal bovine serum (FBS) were obtained from Biological Industries (Cromwell, CT, USA). Mouse monoclonal antibody Anti-C3/C3b and Rabbit polyclonal antibody Anti-OVA were supplied by Abcam (Cambrige, UK) and Rockland Inc (Limerick, PA, USA) respectively. 3,3′,5,5′-Tetramethylbenzidine (TMB) and AlamarBlue® were obtained from Sigma Aldrich (Darmstadt, Germany), Buffer Veronal was obtained from Lonza (Basel, Switzerland) and Cobra Venom Factor was purchased from Quidel (San Diego, CA, USA). 

### 2.2. Development of Nanosystems

For the development of primary nanoemulsions (NEs) we used an experimental design where we studied the influence of TCPH (30, 60, and 120 mg), LP-HCO (25, 50, and 100 mg), and EmFAS-30 (5, 10, and 20 µL) on the physicochemical properties of nanoemulsions. The optimal conditions were obtained after the results were analyzed by Design-Expert 10 (USA).

NE were prepared using the solvent displacement technique [24]. Briefly, different amounts of LP-HCO, TCPH, and EmFAS-30 were dissolved in 750 µL ethanol followed by the addition of 4.25 mL of acetone. This organic phase was immediately poured over 10 mL of water for the formation of the NEs. The organic solvents were eliminated by evaporation under vacuum (Rotavapor Heidolph, Germany) to a constant volume of 5 mL.

The preparation for chitosan nanocapsules in one-step (CSNCs) was the same as the preparation of NE described above, but instead of water, 0.05% *w/v* chitosan solution was used. After the organic solvent evaporation under vacuum, the theoretical concentration of chitosan was 1 mg/mL. For the two-step CSNCs preparation method, we incubated primary NE with CS (0.2% *w*/*v*). The volume ratio used was 1:1 (NE:chitosan solution), the theoretical concentration of chitosan in two-step CSNCs was 1 mg/mL.

CSNCs were isolated by ultracentrifugation (Sorval MX 150+, Thermofiher, Waltham, MA, USA) at 488000 RCF for 1 h at 15 °C. The formulations were then resuspended in the same amount of ultrapure water that was used initially in order to reach a final theoretical polymer concentration of 1 mg/mL.

### 2.3. Physicochemical Characterization of the Nanosystems

The hydrodynamic diameter and polydispersity index of the formulations were determined by Dynamic Light Scattering (DLS) and the zeta potential was measured by laser-Doppler anemometry (Zetasizer^®^, NanoZS, Malvern Instruments, Malvern, UK). For the first measurement, the samples were diluted with ultrapure water and for the second measurement they were diluted with 1 mM KCl. Morphology of the nanosystems was carried out by Scanning Transmission Electron Microscopy (STEM) (Inspect 50, The Netherlands). The samples were stained with 2% (*w*/*v*) phosphotungstic acid solution.

### 2.4. Stability of Nanocapsules upon Physiological/RPMI and Storage Conditions

The stability of the nanocapsules was evaluated by monitoring the evolution of particle size upon incubation in PBS (37 °C, pH = 7.4) for 48 h. In subsequent studies in cells, nanocapsules were incubated in supplemented RPMI and formulation stability was evaluated under the same conditions (37 °C, pH = 7.4 for 48 h) In order to evaluate the stability in storage conditions, the formulation was stored at 4 °C as an aqueous suspension for 14 months. Particle size and superficial charge were analyzed at different times (0.25, 0.5, 1, 2, 3, 4, 5, 6, 8, 10, 12, and 14 months).

### 2.5. Ovalbumin Association to the Nanocapsules 

Ovalbumin (OVA) was used as the model antigen. The association of OVA was performed using isolated nanocapsules (Section 2.2). CSNCs were incubated in equal volumes with different amounts of OVA, in 6:1, 4:1, 2:1, and 1:1 theoretical chitosan:antigen mass ratios. Incubation was performed at room temperature (RT) for 1 h. The OVA-loaded formulations were isolated and the physicochemical properties of loaded nanocapsules were analyzed as previously described (Section 2.2 and Section 2.3).

OVA association efficiency of the 2:1 chitosan: OVA formulation was determined by ELISA. CSNCs with this ratio were isolated and treated with acetonitrile (AcN) in a ratio of 4:5 (AcN:NC) and 100 µL of each of the samples was incubated overnight at 4 °C in a EIA/RIA 96-well plate (Corning, Corning, NY, USA). Plates were washed and blocked with 5% PBS-nonfat milk for 2 h at RT. Rabbit anti-OVA Ab was diluted (1:10,000) and incubated for 2 h at RT. The plates were then washed again and secondary Ab (goat anti-rabbit IgG) was added (diluted to 1:3000) and incubated for 2 h at RT. After this time the plates were washed again 5 times and bound Ab was revealed with by adding 3,3′,5,5′-Tetramethylbenzidine. This incubation then continued for 20 min and was stopped with 50 µL of H_2_SO_4_ 2N. The absorbance was quantified at 450 nm (Stat Fax 2100, Awareness Technology, Ramsey, MN, USA). Each of the samples was tested at least three times.

### 2.6. Study of the Cell Viability of the Nanosystems

Raw 246.7 cells (ATCC, Manassas, VA, USA) were cultured in RPMI supplemented with 5% fetal bovine serum (FBS), 1% (*v/v*) MEM eagle solution, and 1% (*v/v*) penicillin-streptomycin at 37 °C in a humidified atmosphere containing 5% carbon dioxide. The RAW 264.7 cell line was obtained from Dr. Mario Faúndez C. 1 × 10^5^ cells were seeded onto 96-well plates and incubated for 24 h before the assay. After this time, different concentrations (27.23–0.21 mg/mL) of isolated CSNCs or NEs were incubated with the cells. The dilution of the formulations were performed in supplemented RPMI. The concentration of the formulations was calculated by taking the sum of the ingredients that form the nanosystems, and dividing it by the total volume of the formulation. The resulting concentration is expressed in mg/mL.

Cells incubated with Triton X-100 (1:10 dilution in RPMI) were used as the positive control and untreated cells as negative control. After 4 h of incubation, the medium was removed and replaced with fresh RPMI. At 24 h, the medium was eliminated and AlamarBlue® diluted in RPMI was added and incubated for 4 h. The absorbance was quantified at 570 and 600 nm (Cytation 5, Biotek, Winooski, VT, USA). The cell viability was calculated using Equation (1):

(1) $$\%\;\mathrm{Cells}\;\mathrm{Viability}=\frac{\left(\varepsilon \mathrm{OX}\;\right)\lambda_{2}A\lambda_{1}-\left(\varepsilon \mathrm{OX}\right)\lambda_{1}A\lambda_{2}\;\mathrm{samples}}{\left(\varepsilon \mathrm{OX}\right)\lambda_{2}A{}^{\circ}\lambda_{1}-\;\left(\varepsilon \mathrm{OX}\right)\;\lambda_{1}A{}^{\circ}\lambda_{2}\;\mathrm{negative}\;\mathrm{control}}*100$$

λ_1_ = 570 nm, λ_2_ = 600 nm
(εOX) λ_2_ = Molar Extinction coefficient for alamarBlue^®^ at 600 nm: 117,216
(εOX) λ_1_ = Molar Extinction coefficient for alamarBlue^®^ at 570 nm: 80,586
Aλ_1_ = Observed absorbance reading for test well
Aλ_2_ = Observed absorbance reading for test well
A°λ_1_ = Observed absorbance reading for negative control well
A°λ_2_ = Observed absorbance reading for negative control well

### 2.7. Study of Complement Activation by Chitosan Nanocapsules 

The study of the complement cascade activation induced in vitro by CSNCs was performed by analyzing the C3/C3b cleavage products. These products were analyzed using Western blot and following the instructions provided by the Nanotechnology Characterization Laboratory [25]. Human plasma was incubated with equal volumes of veronal buffer (145 mM NaCl and 10 mM barbital, pH 7.4) and the treatment solution. The treatment solutions were different dilutions of CSNCs (2.18–0.54 mg/mL), the positive control (cobra venom factor) or the negative control (dPBS). The CSNCs concentration was determined using the same method that was used in the cell viability assays (Section 2.6). The samples were mixed and incubated at 37 °C for 30 min. 5 µL of each of the samples was loaded on a 10% SDS-PAGE gel and subjected to electrophoresis on a 10% separation gel at 200 V (Bio-Rad, Hercules, CA, USA) and then transferred to a PVDF membrane (Immun-Blot, Biorad; , Hercules, CA, USA). The membranes were then blocked for 1 h at RT (nonfat milk 5% (*w/v*)). The PVDF membranes were incubated overnight with mouse mAb against human C3/C3b (1:2000) at 4 °C. The membranes were then carefully washed and incubated with secondary anti-mouse antibodies (goat anti-mouse IgG, 1:3000) for 1 h at RT. Finally the membranes were revealed using Clarity™ Western ECL Substrate (Chemidoc, Bio-Rad, Hercules, CA, USA); and the results were analyzed using ImageLab.

### 2.8. Transdermal Studies

The skin absorption ability of CSNCs was evaluated in ex vivo experiments using various regions (hind leg, fore leg, and back) from 2–3 day old pig skin [26]. The skin was thawed, and any hair was peeled before each experiment. Franz vertical diffusion cells (area 4.15 cm^2^) were used (Laboratory Glass Apparatus Inc., Berkeley, CA, USA), and the temperature (37 °C) and agitation (300 rpm) were constant throughout the experiment. 

The donor solution was 1 mL OVA loaded-CSNCs or OVA in PBS as control. The amount of OVA was 250 µg for each experiment. The receptor solution was PBS. At 24 h, the receptor solution was placed in an oven (Fisher Isotemp^®^ Oven Senior Model, Germany) at 50 °C until its complete evaporation. Samples were reconstituted in 1 ml of ultrapure water and analyzed by quantitative Western blot [27]. Briefly, samples were reduced with β-mercaptoethanol and loaded onto a 4% acrylamide stacking gel. As a control, different concentrations of OVA (50–2 ng) were treated as described above and loaded onto the same gel. Gel and membranes were treated as described in Section 2.7. The only difference between these procedures are the antibodies used. The primary Ab was rabbit polyclonal antibody Anti-OVA (1:2000) and the secondary Ab was Goat Anti-Rabbit IgG.

The amount of OVA that was retained in the skin was also quantified. At the end of the experiment, the skin was washed with PBS and deposited in 15 mL of methanol for 16 h. The skin was subsequently discarded and the suspension was treated and quantified using the same method that was used for the receptor solution. This procedures was carried out 6 times (*n* = 6).

### 2.9. Data Analysis 

The complement activation and transdermal experiments were both analyzed by *t*-student. Differences were considered significant at a level of *p* < 0.05. 

## 3. Results and Discussion

The aim of this work is the rational design of chitosan nanocapsules (CSNCs) as a carrier for transcutaneous vaccination. For this purpose, we chose nanocapsules because their shell-nucleus structure allows inclusion of active molecules and antigens in the core or attached in their corona [28]. The rational design of nanosystems for transcutaneous delivery requires smaller particles because the size of the carrier is essential. Literature shows that smaller sizes are preferable for transdermal absorption [29]. Although there are chitosan nanocapsules for antigen delivery [30], smaller systems are necessary when considering a transdermal approach. Chitosan was selected as the corona in this experiment because of its proven action as an adjuvant and its penetration enhancer ability [31].

### 3.1. Preparation and Characterization of Small-Sized Chitosan Nanocapsules 

**Figure 1:**
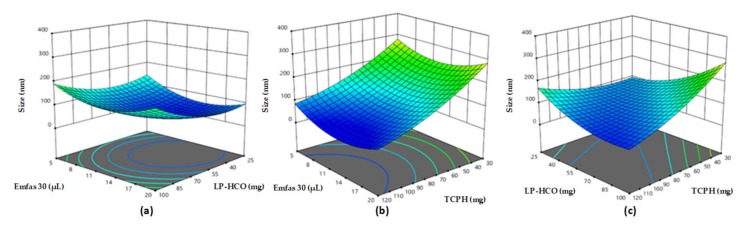
Influence of mass of Lipocol HCO-40 (LP-HCO), vitamin E (TCPH), and the volume of Emulphor FAS-30 (EmFas-30) on the hydrodynamic diameter of nanoemulsiones. (**a**) The amount of TCPH remained constant (60 mg); (**b**) The amount of LP-HCO remained constant (100 mg); (**c**) The amount of EmFas-30 remained constant (20 µL).

Solvent displacement technique was used for the design of CSNCs [24] and the rational design was based on obtaining the smallest size of particles possible that also had an adequate zeta potential. We used an experimental design for the development of a primary nanoemulsion (NE) in order to achieve this. The amounts that were used in this experimental design are detailed in Table S1. The components used are Generally Recognized As Safe (GRAS) and the variables in this design were the mass of Lipocol HCO-40 (LP-HCO), the volume of Emulphor FAS-30 (EmFas-30), and the mass of vitamin E (TCPH). The aqueous phase was always 10 mL of ultrapure water. The TCPH was used as an oily core for its immunoadjuvant properties [32]. Figure 1 shows the influence of these three components on the size of the NEs. The size of the formulations varied between 70 and 200 nm, and TCPH was the component that had the biggest proportional influence on the size of the formulations. The LP-HCO had a slight effect in the size of formulations while the volume of EmFas-30 did not have any effect. Other authors have also noted this relationship between a smaller oil concentration and smaller particle size [33]. The variation of size when altering the concentration of LP-HCO has also been reported and our results are in line with these findings results [34]. 

The zeta potential of NEs varied between −9 and –30 mV, and the excipient that had the biggest effect on superficial charge was LP-HCO (Figure S1). We expected that the variation of EmFas-30, being an anionic surfactant, would have had a greater effect on superficial charge, but this is something that we did not observe. This could be because the concentration interval studied was reduced (5–20 µL) and we expect that a significantly increased concentration of this surfactant also increases the surface charge of the nanosystems. 

When the most adequate amount of the different components for NE formation was determined, the CSNCs were prepared. We used the two methods which were previously described to prepare CSNCs; one-step and two-step. One-step is when the emulsification and chitosan deposition onto the oily nucleus occur at the same time, and two-step is when NEs is formed prior to incubation with an aqueous solution of chitosan. We chose three NEs, from these the prepared our CSNCs using both the one-step and two-step preparation method. All of the three NEs chosen had different sizes and different amounts of TCPH and LP-HCO. The amount of EmFas-30 remained constant for the all formulations (20 µL). Table 1 shows the compositions and physicochemical properties of the different formulations.

The resulting CSNCs exhibited nanometric size and always had a larger size than their respective NEs. The zeta potential was always positive indicating the presence of chitosan on the surface of nanocapsules. The PI was always higher in CSNCs than NEs. This value is acceptable since it indicates that there was only one population of particles. The smallest CSNCs was obtained with NE 1 and prepared using the one step method and was therefore chosen for further studies (stability, OVA association, cell viability, complement activation, and ex vivo studies). Although in the literature there are different formulations of chitosan nanocapsules for diverse applications [35,36,37], the prototype that we present has a size of around 100 nm, making it the smallest that has been found up to now. This is due to the rational design of this formulation which considered application in transcutaneous vaccination. The narrow size distribution that our formulations show ensure antigen association uniformity, stability, and capability [38].

**Figure 2:**
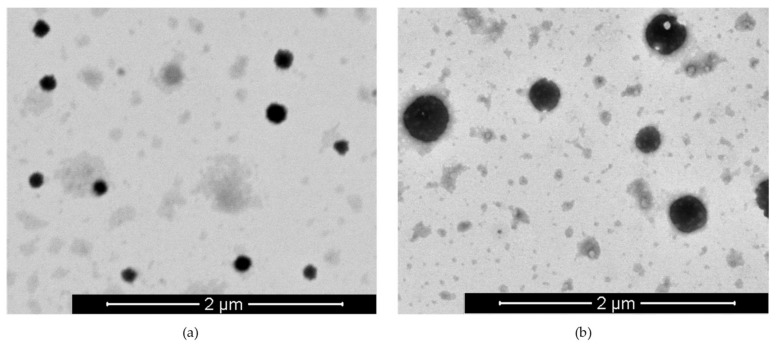
Scanning Transmission Electron Microscopy (STEM) images of blank chitosan nanocapsules (CSNCs) (**a**) and OVA-loaded CSNCs (**b**).

Scanning Transmission Electron Microscopy (STEM) images show that the CSNCs are spherical and homogenous Figure 2a. 

### 3.2. Stability of Chitosan Nanocapsules

**Figure 3:**
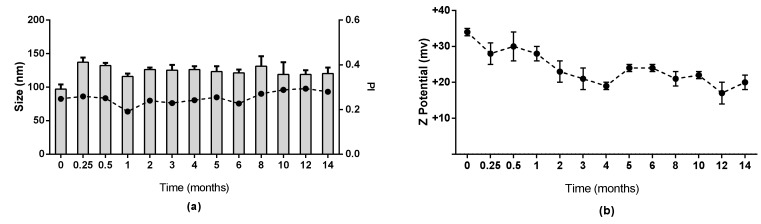
Stability of chitosan nanocapsules during storage at 4 °C for 14 months. (**a**) = size and polydispersity index (PI) (**b**) = zeta potential (mean ± SD, *n* = 3).

The stability of CSNCs was evaluated in physiological conditions and in supplemented RPMI at 37 °C. Results are shown in Figure S2. No significant change in size was observed, indicating stability of the formulation for at least 48 h in these conditions. The formulation was evaluated in storage conditions and CSNCs maintained their physicochemical properties for at least 14 months at 4 °C. These results indicate that this formulation is stable in these conditions and for this amount of time (Figure 3).

### 3.3. Preparation and Characterization of Ovalbumin-Loaded Chitosan Nanocapsules

Ovalbumin (OVA) was used as the model antigen. This protein has a molecular weight of 45 KDa, an isoelectric point of 5.1, and is regularly used in the design of vaccine development studies [39]. We incubated different concentrations of this antigen with isolated CSNCs. The positive charge of the corona facilitates the ionic interaction with the antigen. As shown in Figure S3, the different concentrations of OVA do not affect the nanometric size of the formulation, but it is important to note that the zeta potential was lower when the OVA concentration was higher. This is to be expected since the antigen loaded in the corona shielded the superficial charge of the CSNCs. A 2:1 ratio (CS:OVA proportion) was chosen to quantify the association efficiency of this carrier through ELISA. The association of OVA was high 75 ± 7% (*n* = 6), and it maintained its physicochemical characteristics (Figure S3). Homogenous population and spherical shape was evaluated by STEM images (Figure 2b). The antigen association in the shell of nanocapsules allows a fast recognition by immunocompetent cells and therefore it is expected that it would trigger an efficient immune response. Other nanoformulations have shown similar or lower OVA loading efficiency in comparison to our CSNCs [40,41].

Western blot technique was used for evaluating the integrity of the antigen after being associated to CSNCs. The association method did not affect the OVA structure thus allowing an antigen delivery with intact antigenicity.

### 3.4. Cytotoxicity and Immune System Interaction of Chitosan Nanocapsules

Different concentrations of isolated-CSNCs and their respective NEs were incubated with the macrophage cell line (RAW 264.7) for 4 h. In previous studies, CSNCs and NEs showed that they maintained their size for at least 48 h in experimental conditions (Figure S2). The AlamarBlue® assay is an oxidation-reduction indicator which is based on detection of metabolic activity. This redox indicator changes color and fluoresces as resazurin is reduced to resorufin; the amount of resofurin produced is proportional to the number of viable cells. This assay is slightly more sensitive than MTT assay and is relatively inexpensive [42]. 

**Figure 4:**
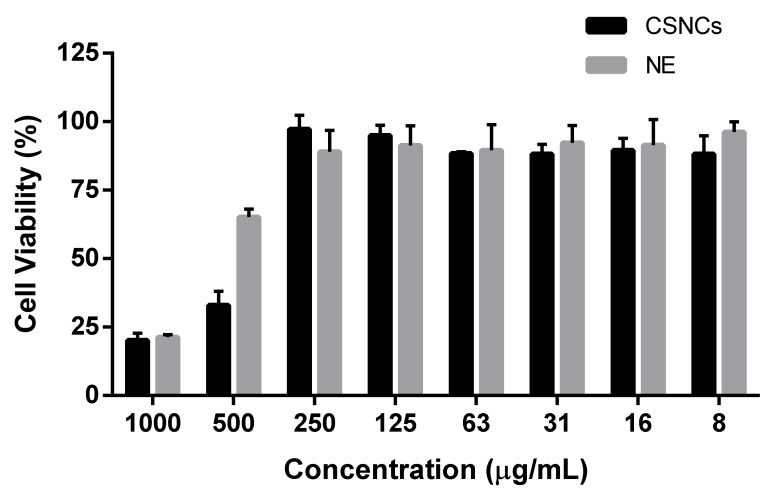
Raw 264.7 cell viability upon 4 h contact with different concentrations of chitosan nanocapsules (CSNCs) or their nanoemulsion (NE). Measurements were taken at 24 h post incubation. (mean ± SD, *n* = 3).

Figure 4 shows the cell viability of both formulations. A wide range of formulation concentration (6.81–0.21 mg/mL) had no effect on cell viability. However at 13.62 mg/mL the cell viability decreased in both formulations and the decrease was more considerable in CSNCs. It is possible that the positive charge of this formulation has a bigger effect on cell viability than the negative charge of the NEs. It should be noted that this same charge gives CSNCs its adjuvant properties, and increases its cellular penetration and bioadhesiveness [43]. At 27.23 mg/mL both formulations showed lower viability on RAW 264.7, however, these high concentrations do not usually occur in in vivo conditions.

**Figure 5:**
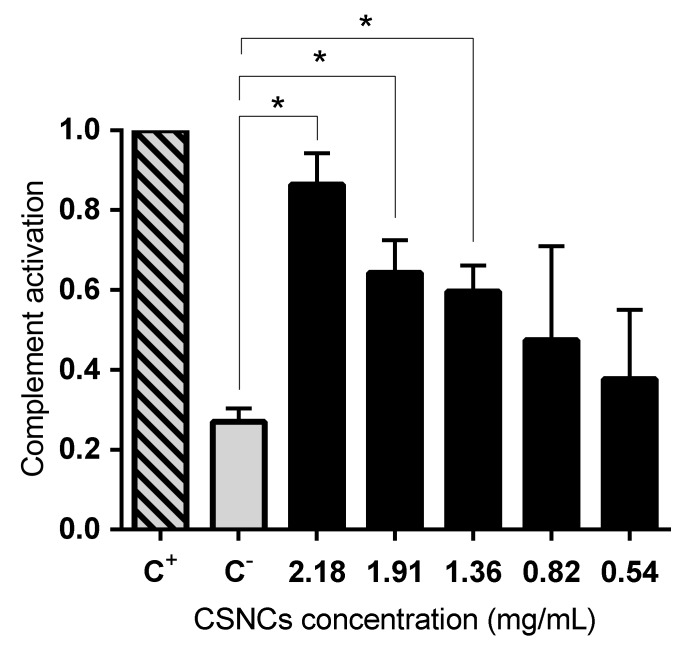
Complement activation of human plasma induced by chitosan nanocapsules (CSNCs). Cobra venom factor (C^+^) and dPBS (C^–^) were used as positive and negative control respectively. The values were normalized by positive control by giving it a value of 1 (mean ± SD, *n* = 3) *p* > 0.05.

In addition to studying the interaction with immune system cells, we studied the activation of the complement cascade caused by CSNCs. The complement system consists of more than 50 plasma and cell surface proteins, which are organized to form three independent, but interactive, activation pathways [44]. The degradation of C3 is present in all three pathways, therefore the presence of this factor indicates the activation of the complement. The results are expressed relative to the positive control (Cobra venom factor). Significant differences were obtained when comparing different CSNCs concentrations with the negative control (dPBS). Figure 5 shows that CSNCs induced complement activation across the wide range of concentrations studied; with significant differences in the highest concentrations studied (2.18, 1.91, and 1.36 mg/mL). The amino groups present in chitosan could be responsible for the complement activation that occurs. The positive charge is capable of inducing a nucleophilic attack on the α-chain of C3b [45]. Complement activation is a useful tool to determine the adjuvant properties of a compound, because complement activation plays a fundamental role in the defense and antigen-specific response. In the specific case of the skin, some fragments of complement C3 have been studied as adjuvant molecules in transcutaneous vaccination [46]. These adjuvant molecules enhance antibody formation. In addition, aluminium salts are the only adjuvants approved for human use in most countries and they also activate the three complement pathways. This characteristic of aluminium salts is related to its adjuvant activity [47]. Similarly, we hope that the formulation developed here has a high interaction with the immune system and that is shows adjuvant activity when it is administered in vivo. Finally, recent studies have shown that the polymeric shell in different types of nanocapsules can influence complement activation and immune system interactions and can also influence in vivo performance [48].

### 3.5. Ex Vivo Transdermal Studies

Ex vivo experiments allow us to observe the transport of the model antigen through the skin and its permanence in it. The skin used was 2–3 day old porcine (pig) skin because the stratum corneum is similar in terms of thickness (near to 21–26 µm) and hair follicle density [26]. Franz-type diffusion cells were used for this purpose and the temperature and agitation remained constant throughout the experiment (24 h). The CSNCs used in ex vivo experiments had a 2:1 ratio of chitosan:OVA. This ratio of chitosan to OVA was used because of its physicochemical characteristics and its high association efficiency (Section 3.3). 

**Figure 6:**
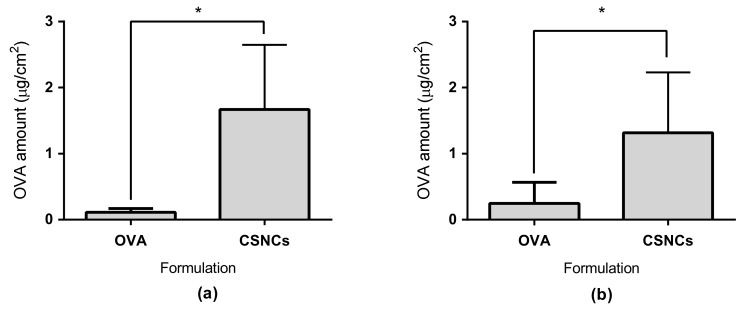
Ex vivo ovalbumin (OVA) transdermal penetration studies. (**a**) Amount of OVA absorbed through the skin after 24 h; (**b**) Amount of OVA retained in the skin after 24 h of experiment. CSNCs = chitosan nanocapsules. Values shown are mean ± SD (*n* = 6). * statistically significant *p* ≤ 0.05.

The antigen associated to small-CSNCs had a higher penetration than OVA in solution (Figure 6a). Moreover, CSNCs enhanced the penetration of OVA by 15 times when compared to OVA in solution (*p* ≤ 0.05). This is a great achievement since the molecular weight of OVA (~43 KDa) is much higher than the 500 Da limit that the transdermal route has for the transport of drugs and antigens [49]. This promoting effect could occur for two reasons. The first reason is that the small size of our nanocapsules allows a higher penetration of the SC because the antigen diffuses through more than one route, including the transcellular and intercellular pathways, and hair follicles. This phenomenon is not something that is observed in delivery systems of a bigger particle size [50]. The second reason is the use of chitosan as a polymeric shell. This polymer has shown the ability to open tight junctions when applied to a mucous membrane. Furthermore, chitosan has shown increased permeation through the skin when administered transcutaneously [51,52]. Additionally, the positive superficial charge of CSNCs given by chitosan could be another factor that further increases the penetration of OVA through the skin [53]. 

The amount of OVA retained in the skin was also quantified. Figure 6b shows that CSNCs retained a larger amount of the model antigen when compared with OVA in solution. We have to keep in mind the high density of immune cells in the skin (especially in the dermis and epidermis), such as epidermal Langerhans and dermal dendritic cells. Another important fact to consider is that that the developed CSNCs activates the complement and could establish a microenvironment for the maturation of APC and a successful transdermal vaccination [54]. Moreover, the CSNCs designed in this study could have advantages such as increased antigen stability; release of antigen in a controlled manner, and synergistic function as an adjuvant; all of which are important factors for transcutaneous antigen delivery [55].

## 4. Conclusions

In this study, we have designed and developed chitosan nanocapsules of a smaller size than other studies and have tested its efficiency in transcutaneous antigen delivery. This nanosystem has a nanometric size of around 100 nm, a positive superficial charge, and a spherical shape. Additionally, these new nanocapsules have shown high stability in different mediums and storage conditions along with a marked association of OVA as the model antigen. They have also shown a capacity to stimulate the immune system with an adequate cell viability profile. The CSNCs developed also promote transdermal penetration of OVA and increase its retention in the skin. Therefore, CSNCs are a feasible platform for transcutaneous vaccination and this kind of carrier could play a part in the development of a more efficient vaccine and contribute to immunization coverage.

## Figures and Tables

**Table 1:** Composition and physicochemical characteristics of nanoemulsions (NE) and chitosan nanocapsules (CSNCs). TCPH: vitamin E, LP-HCO: Lipocol HCO-40, and PI: polydispersity index. The amount of EmFas-30 remained constant (20 µL).

Formulation	TCPH (mg)	LP-HCO (mg)	Size (nm)	PI	ζPotential (mV)
NE 1	60	50	80 ± 2	0.175	−27 ± 2
CSNCs (two-step)			145 ± 20	0.381	+32 ± 3
CSNCs (one step)			97 ± 7	0.248	+36 ± 2
NE 2	120	50	130 ± 3	0.129	−27 ± 8
CSNCs (two-step)			235 ± 31	0.257	+38 ± 3
CSNCs (one step)			192 ± 17	0.302	+38 ± 1
NE 3	120	100	167 ± 3	0.141	−25 ± 2
CSNCs (two-step)			384 ± 25	0.241	+34 ± 8
CSNCs (one step)			194 ± 12	0.280	+32 ± 2

## Supplementary Materials

The following are available online at http://www.mdpi.com/2079-4991/8/9/659/s1, Table S1 shows the variables and amount in the experimental design. Figure S1 shows the influence of mass of Lipocol HCO-40 (LP-HCO), Vitamin E (TCPH) and the volume of Emulphor FAS-30 (EmFas-30) on the zeta potential of nanoemulsions. Figure S2 shows Stability of chitosan nanocapsules in physiological (PBS) and media (RPMI) conditions (37 °C and Ph = 7.4) for 48 h and Figure S3 shows the physicochemical properties of blank chitosan nanocapsules (CSNCs) and OVA-loaded chitosan nanocapsules (CSNCs:OVA) in different proportion (1:1; 2:1; 4:1; 6:1 chitosan:OVA mass ratio).
