# Supplementary material for: Lower-Sized Chitosan Nanocapsules for Transcutaneous Antigen Delivery
Source: Nanomaterials (Basel). 2018 Aug 26;8(9):659. doi: 10.3390/nano8090659 (PMC6164329; doi:10.3390/nano8090659)
Supplement: Supplementary file 1 [file nanomaterials-08-00659-s001.pdf]

nanomaterials

Supplementary Materials

Table S1: Component and amount used in the experimental design.

Component	Function	Composition		
Vitamin E (mg)	oil	30	60	120
Lipocol HCO-40 (mg)	Surfactant	25	50	100
Emulphor FAS-30 (µL)		5	10	20

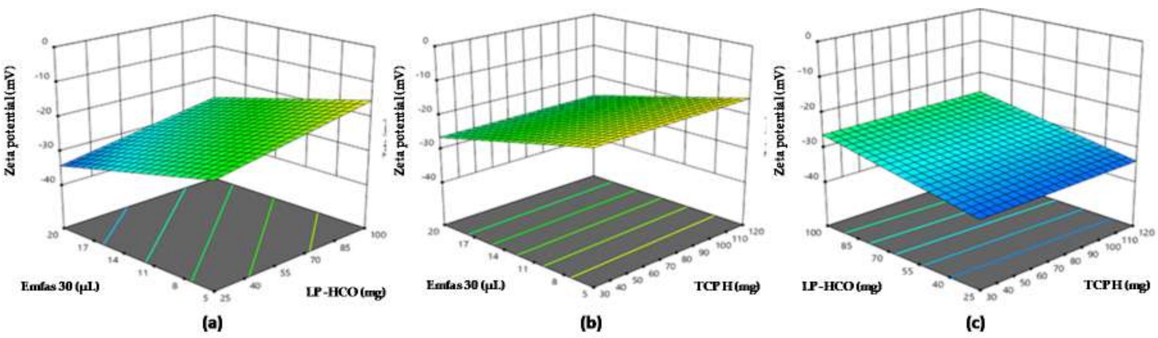

Figure S1: Influence of mass of Lipocol HCO-40 (LP-HCO), Vitamin E (TCPH) and the volume of Emulphor FAS-30 (EmFas-30) on the zeta potential of nanoemulsiones. (a): The amount of TCPH remained constant (60 mg). (b): The amount of LP-HCO remained constant (100 mg). (c): The amount of EmFas-30 remained constant (20 µl).

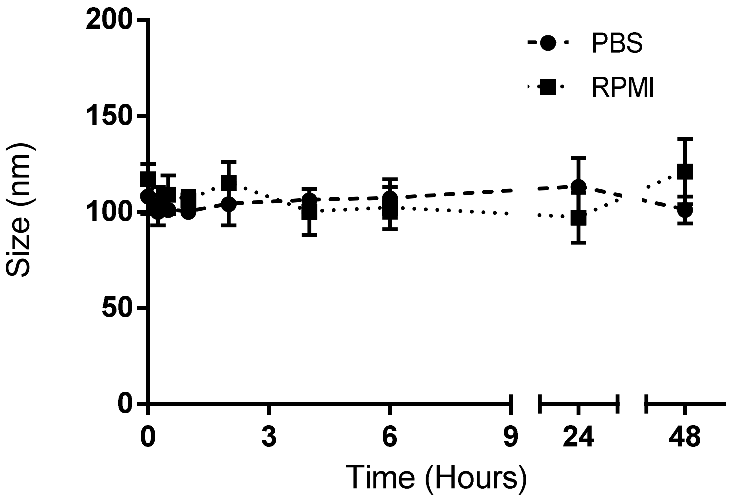

Figure S2: Stability of chitosan nanocapsules in physiological (PBS) and media (supplemented RPMI) conditions. (37°C and pH=7.4) for 48 h. (mean ± SD, n=3).

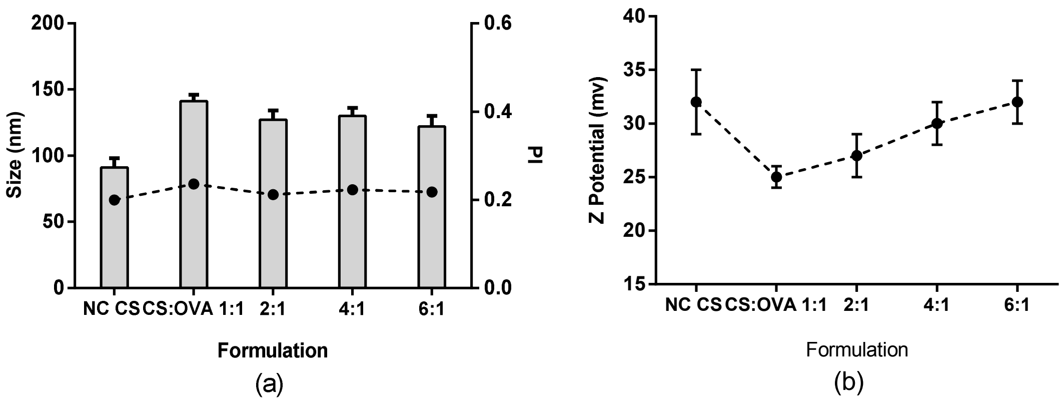

Figure S3: Physicochemical properties of blank chitosan nanocapsules (CS NC) and OVA-loaded chitosan nanocapsules (CS NC:OVA) in different proportions (1:1; 2:1; 4:1; 6:1 chitosan:OVA mass ratio). A: Size and polydispersity index (PI) and B= zeta potential.
